# Supplementary material for: Foxh1 is a locus-specific PRC2 recruiter governing germ layer silencing
Source: bioRxiv. 2025 Sep 22:2025.09.21.677640. Preprint. [Version 1] doi: 10.1101/2025.09.21.677640 (PMC12485744; doi:10.1101/2025.09.21.677640)

bioRxiv preprint doi: https://doi.org/10.1101/2025.09.21.677640; this version posted September 22, 2025. The copyright holder for this preprint (which was not certified by peer review) is the author/funder, who has granted bioRxiv a license to display the preprint in perpetuity. It is made available under aCC-BY 4.0 International license.

Supplementary Figure Legends

Supplementary Figure 1: Temporal expression patterns of candidate transcription factors involved in Ezh2 recruitment to enhancers, and GG term enrichment of genes potentially regulated by Ezh2. (A). Temporal expression profiles of *ezh1* and *ezh2*. (B) GO term analysis of genes associated with Foxh1 and Ezh2 binding. (C-E) Temporal expression profiles of *gcm1/gcm2*, *zrb10/zrb11*, and the *e2f* gene family. (F) GO term analysis of genes associated with Foxh1-independent Ezh2 peaks.

Supplementary Figure 2: Location of indel mutations generated by CRISPR/Cas9 mutagenesis. A) Genomic PCR and Sanger sequencing used for F1 genotyping.
DNA from individual F1 embryos, generated by crossing a Foxh1 CRISPR F0 female with a wild type male, was amplified by PCR and sequenced. The germline of CRISPR F0 female carried 4 types of indel mutations, with no wild type *foxh1* sequences detected, indicating that nearly all germ line cells harbored these mutations. (B) Predicted mutant Foxh1 proteins resulting from the indels identified in (A). (C) Temporal gene expression profiles of *zic* family genes.

Supplementary Figure 3: Quantification of H3K27me3 deposition profiles at ectodermally and endodermally expressed genes, measured in isolated ectoderm and endoderm explants from wild-type and M*foxh1* embryos. ‘Upstream,’ ‘gene body,’ and ‘downstream’ correspond to the 20 kb regions upstream of the TSS, across the gene body, and 20 kb downstream of the TES, respectively. Values shown represent average RPKM values over the designated region.

Supplemental Figure 1

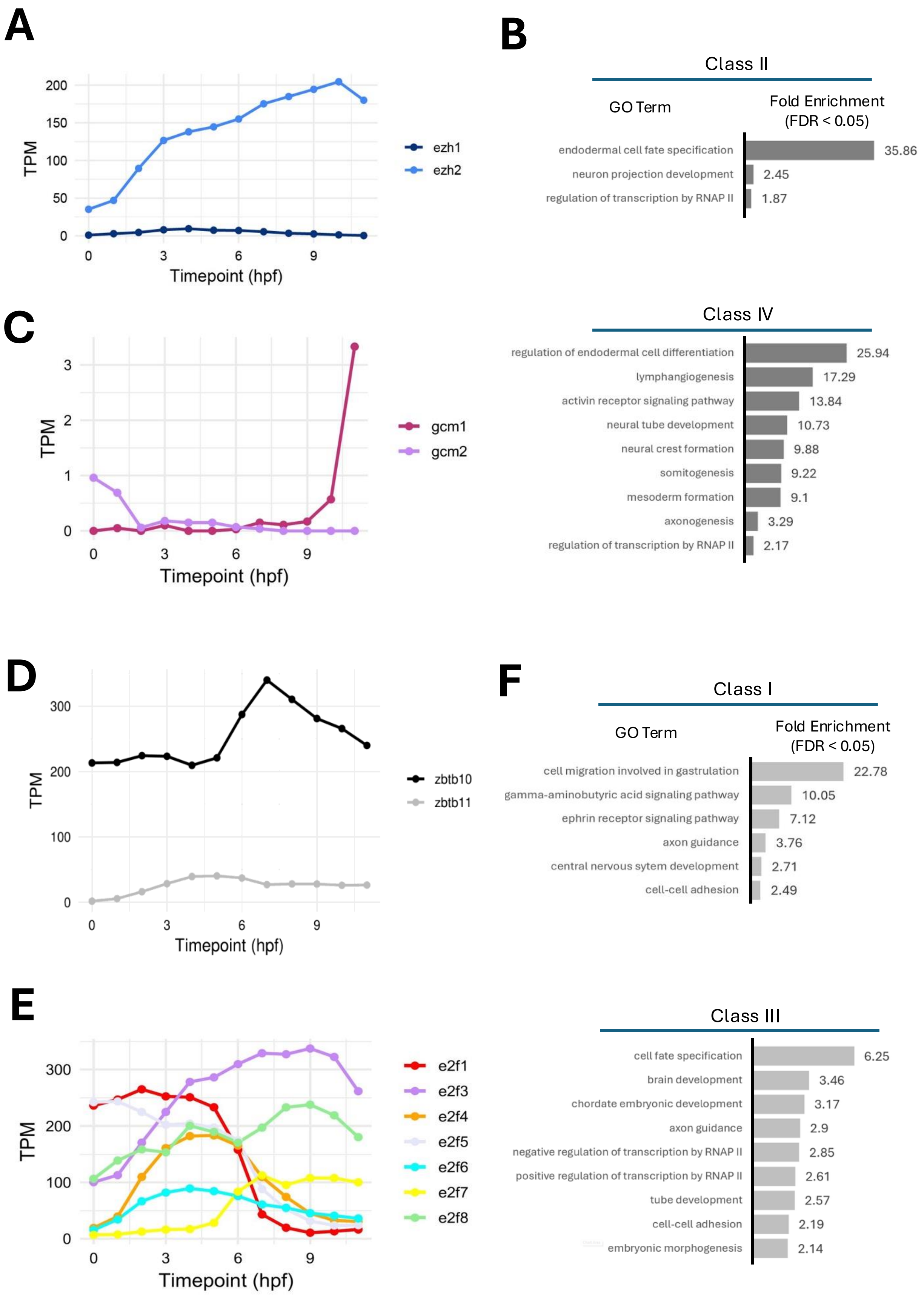

Supplemental Figure 2

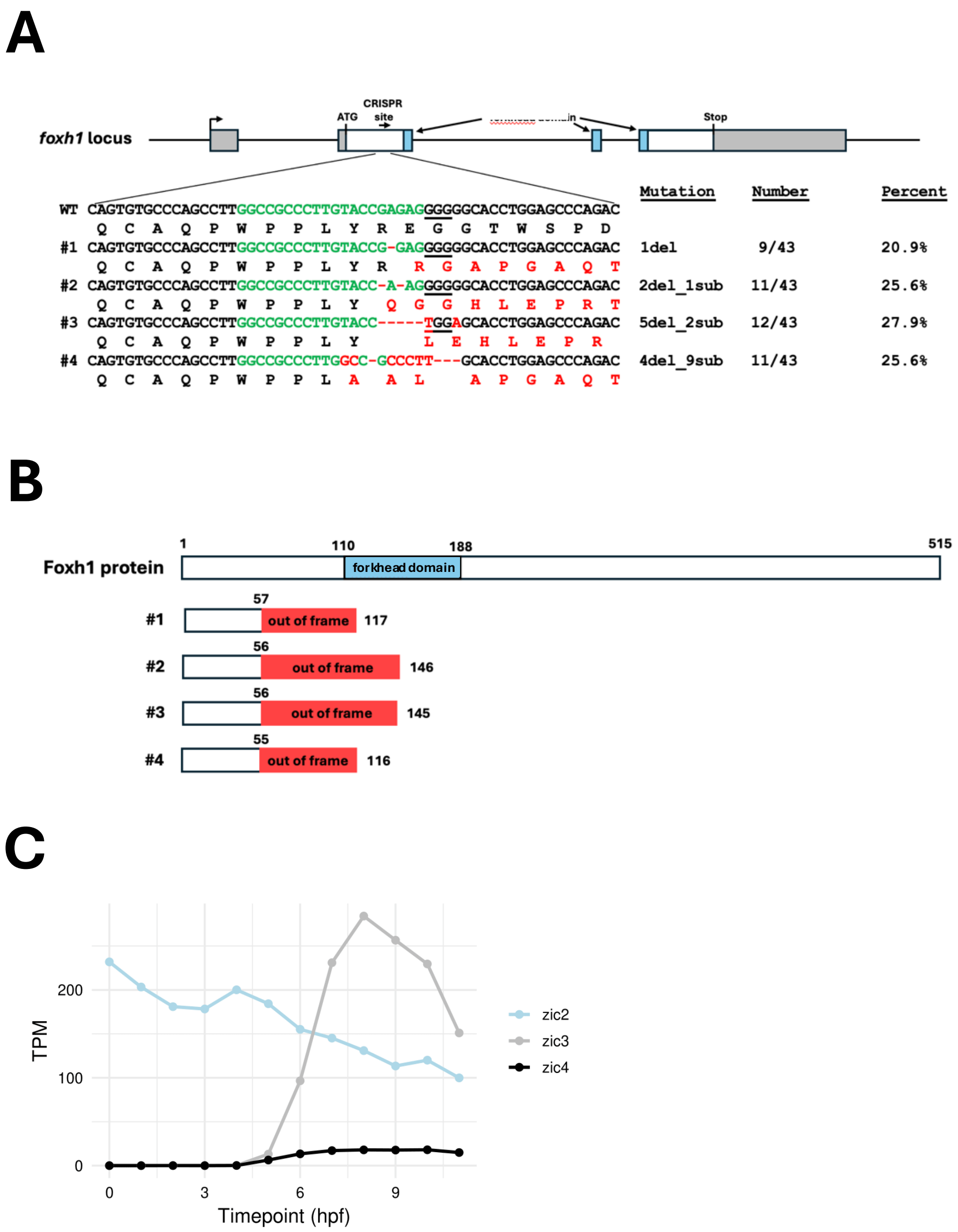

Supplemental Figure 3

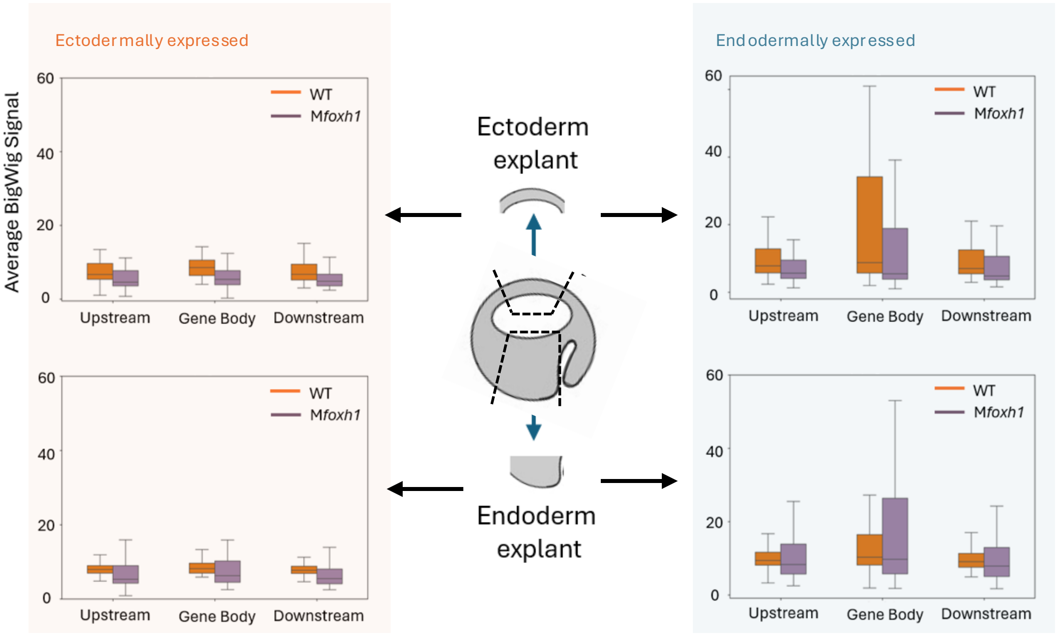

Supplement: Supplement 1 [file NIHPP2025.09.21.677640v1-supplement-1.pdf]
